# A behaviour and disease transmission model: incorporating the Health Belief Model for human behaviour into a simple transmission model

**DOI:** 10.1098/rsif.2024.0038

**Published:** 2024-06-05

**Authors:** Matthew Ryan, Emily Brindal, Mick Roberts, Roslyn I. Hickson

**Affiliations:** ^1^ Commonwealth Scientific and Industrial Research Organisation (CSIRO), Adelaide, Australia; ^2^ Australian Institute of Tropical Health and Medicine, James Cook University, Townsville, Australia; ^3^ New Zealand Institute for Advanced Study, Massey University, Auckland, New Zealand

**Keywords:** behaviour modelling, epidemiology, transmission modelling, Health Belief Model

## Abstract

The health and economic impacts of infectious diseases such as COVID-19 affect all levels of a community from the individual to the governing bodies. However, the spread of an infectious disease is intricately linked to the behaviour of the people within a community since crowd behaviour affects individual human behaviour, while human behaviour affects infection spread, and infection spread affects human behaviour. Capturing these feedback loops of behaviour and infection is a well-known challenge in infectious disease modelling. Here, we investigate the interface of behavioural science theory and infectious disease modelling to explore behaviour and disease (BaD) transmission models. Specifically, we incorporate a visible protective behaviour into the susceptible–infectious–recovered–susceptible (SIRS) transmission model using the socio-psychological Health Belief Model to motivate behavioural uptake and abandonment. We characterize the mathematical thresholds for BaD emergence in the BaD SIRS model and the feasible steady states. We also explore, under different infectious disease scenarios, the effects of a fully protective behaviour on long-term disease prevalence in a community, and describe how BaD modelling can investigate non-pharmaceutical interventions that target-specific components of the Health Belief Model. This transdisciplinary BaD modelling approach may reduce the health and economic impacts of future epidemics.

## 1. Introduction

Understanding how an infection spreads throughout a community is vital for preparedness by governments, companies and individuals to reduce the health and economic challenges that might arise. A key component of disease spread is the human factor because behaviour can determine how quickly or slowly we might expect an infection to be transmitted through populations, as evidenced by the recent COVID-19 pandemic [1–4]. For example, how individuals in the community adopt health-protective behaviours (such as facemask wearing and handwashing) can help to reduce transmission, whereas individuals engaging in risky behaviour (such as ‘COVID-19 parties’) can potentially increase disease transmission. In spite of this, many epidemiological models either do not explicitly account for human behaviour or consider behaviour that is not justified by behavioural sciences theory [5]. By failing to account for human behaviour appropriately in epidemiological modelling, ‘business-as-usual’ forecasts of an epidemic can be inaccurate, which opens the door for criticism and distrust from the public [6]. This reduced trust can then have significant follow-on effects, since trust in institutions is important for engagement in protective public health measures [7].

The call for incorporating human behaviour into epidemiological models is well known [8], and there is a community of active research trying to answer that call (see for example Bedson *et al*. [9] for a recent review). One of the key challenges in appropriately capturing human behaviour is one of cross-disciplinary communication [10]. This is highlighted in a recent review of agent-based epidemiological models accounting for human behaviour, where Weston *et al*. [5] found that only five of the 42 papers reviewed explicitly mentioned behavioural science theory to justify behavioural uptake. Another challenge is that models that describe human behaviour are complex, various and do not lend themselves to mathematical modelling. Many psychological models focus on mediating and moderating effects that partially explain a key behaviour, rather than easily translatable models. Finally, when human behaviour models are considered, data availability around how and why humans perform behaviours in response to an epidemic are scarce [9,10]; more work is needed to conceptualize and explain mass behaviours to understand those critical in response to an epidemic (see Brindal *et al*. [11]).

There are many different approaches that have been used to incorporate aspects of human behaviour in infection transmission modelling. Broadly, these fall into agent-based modelling, network theory, evolutionary game theory and stratified compartmental modelling approaches. Agent-based modelling is often used to capture the transient and individual nature of behaviour [5,9,12] and how this interacts with infectious diseases. Network theory approaches explicitly model contact network structure in the population and the dual dynamics of behaviour and infection across these networks [13–16]. Evolutionary game theory models contact dynamics through cost–benefit analysis averaged over the population [17,18]. Stratified compartmental models investigate new compartments for individuals performing a given behaviour and explore the population-level dynamics of both behaviour and infection [19–22]. We focus on stratified compartmental modelling approaches for the population-level insights they offer.

In compartmental models, heterogeneity in population behaviour is usually captured by additional compartments in the model. For example, in human immunodeficiency virus modelling, it is common to include separate compartments for individuals with high and low sexual activity as their risk of infection is different. There have also been models that allow dynamic behaviour change that can evolve with the evolution of the epidemic. Funk *et al*. [19] propose additional compartments for susceptible, infectious and recovered individuals to capture ‘fear’ in the population that causes them to perform health-protective measures and reduce the risk of their infection; Agaba *et al*. [20] recently extended this model to have more complex behaviour transitions. In a similar approach, Perra & Vespignani [21] consider a fear compartment of susceptible individuals to reduce disease risk, where fear may be transmitted through either social influence (interacting with individuals with fear of the disease) or through contact with the disease. De Valle *et al*. [22] consider a health-protective behaviour for susceptible and infectious individuals that is taken up at a constant rate during a given time period to capture behaviour becoming present once an infection is prevalent in the community. However, although many of the behaviours these authors consider are of interest (for example, fear has been widely considered in behavioural theory and public health, although with inconsistent effects [23]), none of these models incorporate psychological justification for their behavioural uptakes.

Here, we explore the interface of behaviour and infection transmission modelling by coupling conventional epidemiological models with theories from behavioural science into a single framework. By way of an example, we investigate an endemic transmission model for a respiratory illness where individuals can take up a protective health behaviour (such as facemask wearing, handwashing or physical distancing) that can reduce the transmission rate of the infection. In particular, we consider the dynamics of a susceptible–infectious–recovered–susceptible (SIRS) compartmental model with protective health behaviour dynamics justified by the socio-psychological Health Belief Model [24,25]. We present theoretical results on the threshold conditions and steady states of the model and discuss how different drivers of the Health Belief Model affect different steady states. We also demonstrate how our model can be used to investigate the effects of health behaviours for reducing disease endemicity, and how we can use this framework to investigate the impact of targeted non-pharmaceutical interventions on infection prevalence.

## 2. The model

The behaviour and disease (BaD) transmission modelling framework looks at modelling infectious diseases in the presence of non-mandated behaviour, where uptake and abandonment of behaviour are motivated by theories from behavioural science. It is important to distinguish non-mandated behaviours from mandated behaviour (such as lockdowns) because mandated behaviours are likely to be influenced by separate psychological factors, such as reactance [26]. Thus, BaD transmission models can be applied to a large variety of different pathogens and modelling structures where behaviour can affect transmission. To explore this framework, we consider a simple compartmental model for a respiratory transmitted infection and explain how to generalize this to a BaD model.

Recall the standard SIRS model for a respiratory transmitted illness where susceptibles (*S*) can become infectious ( I ) before becoming recovered ( R ), with a waning immunity where recovered individuals become susceptible again. Let *S*, I and R denote the proportion of susceptible, infectious and recovered individuals, respectively, such that S+I+R=1 . We assume an isolated community and ignore human movement and demography. Then, the SIRS model is governed by the following set of differential equations:


(2.1) $$\begin{array}{rl} \dot{S} & =-\lambda S+\nu R\;, \end{array}$$

(2.2) $$\begin{array}{rl} \dot{I} & =\lambda S-\gamma I\;, \end{array}$$

(2.3) $$\begin{array}{rl} \dot{R} & =\gamma I-\nu R\;, \end{array}$$

where $\dot{X}=\frac{dX}{dt}$ for each X=S,I,R , λ is the force of infection, $\gamma^{-1}$ is the average infectious period and $\nu^{-1}$ is the average immune period (table 1).

**Table 1.** Parameters for the BaD SIRS transmission model. The left-aligned parameters are functions of the indented ones where applicable. All parameters and variables specified with greek letters have dimension time^−1^. Other parameters and state variables are dimensionless.

parameter	description	value(s)
λ(t)	the force of infection	
β	the transmission rate, obtained from reasonable estimates of the disease characteristic	{1.5, 8.2}
c	the efficacy of behaviour B in reducing susceptibility	{0, 0.5, 1}
p	the efficacy of behaviour B in reducing infectiousness	{0, 0.5, 1}
γ	the recovery rate such that $\gamma^{-1}$ is the average infectious period	1
ν	the rate of waning immunity such that $\nu^{-1}$ is the average immune period	40
ω(t)	the ‘force of infection’ for behaviour uptake	
$\omega_{1}$	the ‘infectiousness’ of behaviour on behaviour	0.4
$\omega_{2}$	the ‘fear of disease’ of infected people on behaviour uptake	8
$\omega_{3}$	constant rate of spontaneous behaviour uptake	0.2
α(t)	the ‘force of infection’ for behaviour abandonment	
$\alpha_{1}$	the ‘infectiousness’ of no behaviour on behaviour abandonment	1.25
$\alpha_{2}$	constant rate of spontaneous behaviour abandonment	0.6

Suppose we introduce a visually perceptible protective health behaviour B (henceforth, referred to as ‘behaviour’ or ‘health behaviour’) into the population that individuals can choose to practise. We model this by stratifying the S , I and R components into $X_{N}$ and $X_{B}$ , X∈{S,I,R} . The strata $X_{N}$ represents the proportion of X who are not performing the behaviour, and the strata $X_{B}$ represents the proportion X who are performing the behaviour. Note that, even though individuals in R are immune to infection, we consider the behavioural stratification of R as these individuals will contribute to the social influence of the behaviour. As we are dealing with proportions of the population, we have


$$\sum_{X\in \{S,I,R\}}(X_{N}+X_{B})=1\;.$$

**Figure 1.**
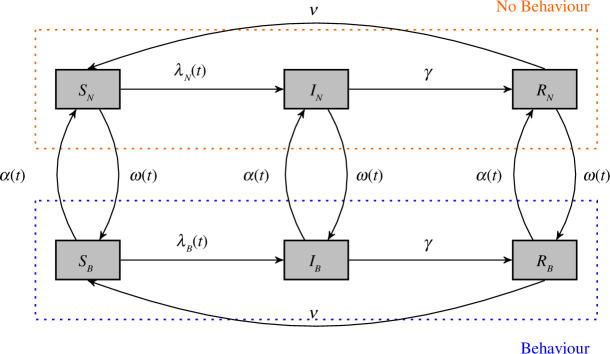
A BaD SIRS model without demography. The parameters are defined in table 1.

The BaD SIRS transmission model is visualized in figure 1. Observe that the transitions between epidemiological states follow the standard SIRS structure, but we now allow behavioural transitions governed by uptake ( ω(t) ) and abandonment ( α(t) ). Having the behavioural transitions evolve dynamically allows us to capture more transient properties of behaviour throughout an epidemic. Note that we assume the behavioural transition rates α(t) and ω(t) are independent of epidemiological state X . This is a simplifying assumption that could be relaxed in future work (see electronic supplementary material).

The BaD SIRS model (figure 1) is described by six differential equations, where the dot represents the derivative with respect to time t . All behavioural and epidemiological parameters are defined in table 1.


(2.4) $$\begin{array}{rl} {\dot{S}}_{N}(t) & =-\lambda_{N}(t)S_{N}(t)+\nu R_{N}(t)+\alpha (t)S_{B}(t)-\omega (t)S_{N}(t)\;, \end{array}$$

(2.5) $$\begin{array}{rl} {\dot{I}}_{N}(t) & =\lambda_{N}(t)S_{N}(t)-\gamma I_{N}(t)+\alpha (t)I_{B}(t)-\omega (t)I_{N}(t)\;, \end{array}$$

(2.6) $$\begin{array}{rl} {\dot{R}}_{N}(t) & =\gamma I_{N}(t)-\nu R_{N}(t)+\alpha (t)R_{B}(t)-\omega (t)R_{N}(t)\;, \end{array}$$

(2.7) $$\begin{array}{rl} {\dot{S}}_{B}(t) & =-\lambda_{B}(t)S_{B}(t)+\nu R_{B}(t)-\alpha (t)S_{B}(t)+\omega (t)S_{N}(t)\;, \end{array}$$

(2.8) $$\begin{array}{rl} {\dot{I}}_{B}(t) & =\lambda_{B}(t)S_{B}(t)-\gamma I_{B}(t)-\alpha (t)I_{B}(t)+\omega (t)I_{N}(t)\;, \end{array}$$

(2.9) $$\begin{array}{rl} {\dot{R}}_{B}(t) & =\gamma I_{B}(t)-\nu R_{B}(t)-\alpha (t)R_{B}(t)+\omega (t)R_{N}(t)\;. \end{array}$$

Assuming homogeneous mixing of the population, we use the frequency-dependent force of infections given by


$$\begin{array}{rl} \lambda_{N}(t) & =\beta \left(I_{N}(t)+(1-p)I_{B}(t)\right),\text{ and}\\ \lambda_{B}(t) & =\beta (1-c)\left(I_{N}(t)+(1-p)I_{B}(t)\right)\\ \; & =(1-c)\lambda_{N}(t). \end{array}$$

When discussing the *behavioural states*—depicted as the various strata, with those practising the behaviour ( B ) in the blue rectangle, and those not ( N ) in the orange rectangle in figure 1—of the model, we will commonly write


$$\begin{array}{rl} N(t) & =S_{N}(t)+I_{N}(t)+R_{N}(t),\;and;\\ B(t) & =S_{B}(t)+I_{B}(t)+R_{B}(t). \end{array}$$

Given that N+B=1 , we can consider the change in proportion of those practising the behaviour or not with the governing differential equations


$$\begin{array}{rl} \dot{N}(t) & =-\omega (t)N(t)+\alpha (t)B(t),\;and;\\ \dot{B}(t) & =\omega (t)N(t)-\alpha (t)B(t). \end{array}$$

This demonstrates that the practice of the behaviour has an SIS-like structure in the sense that ‘susceptible’ individuals ( N ) can become ‘infected’ with the behaviour ( B ) and eventually ‘recover’ back to ‘susceptible’. What remains is to define the behavioural transitions ω(t) and α(t) , which we do by incorporating theory from the behavioural sciences.

### 2.1. The Health Belief Model

Many theories from behavioural science have been applied in the context of infectious diseases and risk assessment [5,27]. The choice of behavioural model is commonly driven by the intent of the study [28], with the most cited models for infectious diseases being the Theory of Planned Behaviour, Protection Motivation Theory, and the Health Belief Model [5,27]. The Theory of Planned Behaviour describes how the beliefs of a person influence their planned behaviours such as hand hygiene or participating in infection screening [29] with a strong focus on intention as the primary predictor of actual behaviour. Protection Motivation Theory aims to capture how an individual will weigh up the costs and benefits of a protective behaviour in response to an appeal to their fears, with a strong focus on cognitive appraisals of perceived threat and ability to manage. It has been applied to investigate preventative, avoidant, and management health behaviours in response to respiratory diseases [30,31]. Although these models have their appeal in the context of infectious diseases, we focus on the Health Belief Model as it is the most commonly used in mathematical modelling of infectious diseases [5]. It combines appraisal and efficacy elements of both behavioural models to predict likelihood of engaging in a health behaviour.

**Figure 2.**
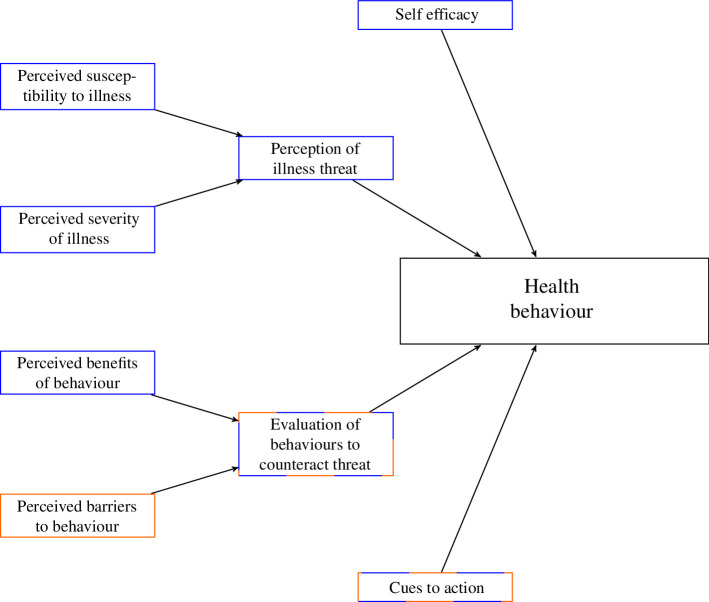
The Health Belief Model [24,25], adapted from Brailsford *et al.* [32]. The blue boxes are the incentives for performing a given health behaviour, and the orange boxes are the barriers that prevent an individual from performing the behaviour. The boxes with both colours can both positively and negatively affect whether someone performs the health behaviour.

The Health Belief Model [24,25] (figure 2) is a socio-psychological behavioural model that aims to describe the factors that contribute towards an individual performing a protective health behaviour, such as wearing a mask or getting vaccinated. The original model considered the perception of illness threat and evaluation of behaviours, but the revised Health Belief Model includes the following six factors (where perception of illness threat and evaluation of behaviours have been divided into two subfactors each) [33]:

— Self-efficacy: confidence in one’s ability to perform the behaviour.
— Perception of illness threat: this captures both perceived susceptibility to the illness and perceived severity of the illness. It is divided into two parts:
  - *Perceived susceptibility*: beliefs about the chance of getting infected.
  - *Perceived severity*: beliefs about the seriousness of the condition and the consequences.
— Evaluation of behaviours to counteract threat: this captures the perceived benefits of the behaviour as well as the perceived barriers to performing the behaviour. It is divided into two parts:
  - *Perceived benefits*: beliefs about the effectiveness of the behaviour to reduce the seriousness of the health threat.
  - *Perceived barriers*: beliefs about material and psychological costs of taking the action.
— Cues to action: external or internal factors that activate the ‘readiness to change’.

The Health Belief Model is the most prominently cited behavioural theory used in an epidemiological context [5,27]. Most notably, Durham & Casman [12] incorporate the Health Belief Model as a decision-making tool in an agent-based model to determine whether their agents will wear a facemask. In their work, Durham & Casman use survey data to consider the perception of illness threat and the evaluation of behaviour outcomes as inputs into a logistic regression model to determine when an agent will take up the health behaviour of mask wearing in a SARS outbreak. However, to simplify their model the authors decouple the dynamics of behaviour and epidemiology.

To the best of our knowledge, the Health Belief Model has not been included in a population-based transmission model. One possible factor for this is that the Health Belief Model is a model for determining why an individual performs a protective health behaviour and is not necessarily used to describe a population-level response. However, basing our behavioural transitions on the Health Belief Model has the advantage that mathematicians and behavioural scientists can work together to translate this modelling to key targets for population interventions and policy to promote positive health behaviour. This includes leveraging existing experimental data as appropriate.

### 2.2. A mathematical model of the Health Belief Model for a visual protective behaviour

We use the Health Belief Model to construct the behavioural transition functions ω(t) and α(t) . To do this, we consider the components of the Health Belief Model that positively influence the health behaviour for the function ω(t) , and those that negatively influence the health behaviour for α(t) (figure 2). From here, we focus our discussion on *visual* health protective behaviours to understand the social-contagion aspect of visual behaviours in the functional forms of ω and α . This captures a wide range of behaviours observed in real-world epidemics such as physical distancing [34], face mask wearing [35] and handwashing [36].

First, consider the rate of behaviour uptake ω(t) . To formulate this in terms of the Health Belief Model, we write


(2.10) $$\omega (t)=f_{self}(t)+f_{susc}(t)+f_{sev}(t)+f_{ben}(t)+f_{cues}(t)\;,$$

where, $f_{self}(t)$ represents the influence of self-efficacy on the rate of behaviour uptake; $f_{susc}(t)$ represents the influence of perceived susceptibility to infection on the rate of behaviour uptake; $f_{sev}(t)$ represents the influence of the perceived severity of the disease on the rate of behaviour uptake; $f_{ben}(t)$ represents the influence of perceived benefits of the behaviour on the rate of behaviour uptake; and $f_{cues}(t)$ represents the influence of cues to action on the rate of behaviour uptake.

Note that equation (2.10) considers the simplifying assumption that each factor of the Health Belief Model is contributing linearly and equally to the transition rate ω(t) . We can relax the assumption that each factor contributes equally, but it will not affect the modelling process as we collect constants of proportionality.

The functional forms of $f_{i}(t)$ , i∈{self,susc,sev,ben,cues} , can be highly complex and depend both on the behaviour and the specific pathogen. For example, if the pathogen has a high mortality rate we may expect the effect of $f_{sev}(t)$ to be large. As a general rule to avoid parameter blowout, we suggest parameterizing each $f_{i}$ around the disease states ( S,I,R ), the behaviour states ( N,B ), the model parameters ( β,γ,ν,p,c ) and time ( t ).

We suggest the following parameterization for the components of ω(t) :

—
$f_{self}(t)\approx$ constant. As we are assuming large numbers in a population-level model, we take $f_{self}$ to represent the average self-efficacy across the population;
—
$f_{susc}(t)\propto I(t)=I_{N}(t)+I_{B}(t)$ . Then, the perceived susceptibility of an individual is proportional to the average number of infectious contacts they have in a time period. Note, this assumes that each individual in the N population is instantaneously aware of the exact prevalence of infection at time t ;
—
$f_{sev}(t)\propto I(t)$ , and hence to the (assumed constant) proportion of infected people with severe cases of the disease (hospitalization or death);
—
$f_{ben}(t)=h_{\omega }(p,c,t)$ , where $h_{\omega }$ is a function describing the public perception of the health behaviours efficacy for both susceptible and infected individuals. Assuming that public knowledge does not change appreciably over the time period of interest, we take $h_{\omega }(p,c,t)=$ constant; and
—
$f_{cues}(t)\propto B(t)+$ constant. We consider the cues to action of the visual protective behaviour to be proportional to the average number of contacts with others adopting the behaviour, as well as a constant source owing to government advertising and signage, and internal cues such as empathy for others.

Combining these assumptions, we can write


(2.11) $$\begin{array}{rl} \omega (t) & =f_{self}(t)+f_{susc}(t)+f_{sev}(t)+f_{ben}(t)+f_{cues}(t)\\ & =s_{1}+s_{2}I(t)+s_{3}I(t)+s_{4}+s_{5}B(t)+s_{6}\\ & =\omega_{1}B(t)+\omega_{2}I(t)+\omega_{3}\;, \end{array}$$

where $\omega_{1}=s_{5}$ captures the social contagion aspect of the visual protective behaviour, $\omega_{2}=s_{2}+s_{3}$ captures the uptake of behaviour owing to fear of the disease, and $\omega_{3}=s_{1}+s_{4}+s_{6}$ captures the uptake of behaviour owing to self-efficacy, benefits of the behaviour and external cues to action. To ensure the model in equations (2.4)–(2.9) are well-defined, we restrict $\omega_{1},\omega_{2}$ and $\omega_{3}$ to be positive and constant over time (table 1).

For the rate of behaviour abandonment α(t) , considering the inhibitors of behaviour uptake (figure 2) gives


$$\alpha \,\left(t\right)=g_{b\,a\,r}\,\left(t\right)+g_{c\,u\,e\,s}\,\left(t\right),$$

where $g_{bar}$ and $g_{cues}$ now describe the influence of perceived barriers to behaviour and cues to action on *maintaining* the health behaviour B . Once again, these are very BaD dependant. For example, when B is the behaviour of facemask wearing and we are considering an airborne pathogen, an aspect of $g_{cues}(t)$ might describe how long an individual feels like they can keep wearing a mask, where positive values would describe abandonment of behaviour owing to exhaustion. Similar to ω(t) , we suggest the following parameterization for α(t) .

—
$g_{bar}(t)=h_{\alpha }(t)$ , where $h_{\alpha }$ is a function describing the public perception of the barriers to the protective behaviour. Assuming (on average) that this perception does not change over the time period of interest, we take $h_{\alpha }(t)=$ constant;
—
$g_{cues}(t)\propto N+$ constant. The cues to action then capture the external cues of visually seeing people *not* performing the behaviour and the internal cues to action such as exhaustion to perform the behaviour.

We may write


(2.12) $$\begin{array}{rl} \alpha (t) & =r_{1}+r_{2}+r_{3}N(t)\\ & =\alpha_{1}N(t)+\alpha_{2}\;, \end{array}$$

where $\alpha_{1}=r_{3}$ captures the social contagion aspect of not performing the visual protective behaviour and $\alpha_{2}=r_{1}+r_{2}$ captures the barriers and cues to action impeding the performance of the health behaviour. Similar to ω , the values $\alpha_{1}$ and $\alpha_{2}$ are assumed to be positive and constant over time (table 1) to ensure that the model is well-defined. Combining equations (2.11) and (2.12) with (2.4)–(2.9) gives the full BaD SIRS transmission model we consider throughout the rest of this article.

## 3. Thresholds and steady states

### 3.1. Threshold conditions

There are three threshold conditions we consider, two that decouple the dynamics of the behaviour and the infection, and one that captures the full dynamics. First, in the absence of behaviour where α=ω=0 , the BaD SIRS transmission model reduces to the classic SIRS model. Considering this situation, we have the *infection reproduction number* or *disease characteristic* given by the well-known


(3.1) $$R_{0}^{D}=\frac{\beta }{\gamma }\;.$$

Second, consider the dynamics of the behaviour in the absence of infection ( I=0 ) given by


$$\dot{B}=\left(\omega_{1}\,B+\omega_{3}\right)\,N-\left(\alpha_{1}\,N+\alpha_{2}\right)\,B.$$

Linearizing this around B=0 gives


$$\dot{B}=(\omega_{1}-(\alpha_{1}+\alpha_{2}))B+\omega_{3},$$

which may be solved to give


(3.2) $$B(t)=\left(B_{0}+\frac{\omega_{3}}{\omega_{1}-(\alpha_{1}+\alpha_{2})}\right)e^{(\omega_{1}-(\alpha_{1}+\alpha_{2}))t}-\frac{\omega_{3}}{\omega_{1}-(\alpha_{1}+\alpha_{2})}\;,$$

where $B_{0}=B(0)$ is the initial condition of the behaviour. This shows that behaviour will always emerge in the system if $\omega_{3}\neq 0$ , but will only spread as a social contagion if


$$\omega_{1}-(\alpha_{1}+\alpha_{2})>0\;.$$

This leads us to the *behavioural reproduction number* or *behavioural characteristic* defined by


(3.3) $$R_{0}^{B}=\frac{\omega_{1}}{\alpha_{1}+\alpha_{2}}\;.$$

Finally, consider the full system (equations (2.4)–(2.9)) with both disease and behaviour present. Here, we calculate the next-generation matrix following Diekmann *et al*. [37] (see the electronic supplementary material for an alternative approach to calculating the next-generation matrix). In equations (2.4)–(2.9), the infection states are $I_{N}$ and $I_{B}$ (we drop the dependence on t for simplicity). The Jacobian of these states is


(3.4) $$\begin{array}{rl} J & =\left[\begin{array}{cc} \beta S_{N}+\frac{\partial \alpha }{\partial I_{N}}I_{B}-\omega -\frac{\partial \omega }{\partial I_{N}}I_{N}-\gamma & (1-p)\beta S_{N}+\alpha +\frac{\partial \alpha }{\partial I_{B}}I_{B}-\frac{\partial \omega }{\partial I_{B}}I_{N}\\ (1-c)\beta S_{B}-\frac{\partial \alpha }{\partial I_{N}}I_{B}+\omega +\frac{\partial \omega }{\partial I_{N}}I_{N} & (1-c)(1-p)\beta S_{B}-\alpha -\frac{\partial \alpha }{\partial I_{B}}I_{B}+\frac{\partial \omega }{\partial I_{B}}I_{N}-\gamma \end{array}\right]\;. \end{array}$$

At the behaviour endemic, infection-free steady state, $S_{N}=N^{*}$ and $S_{B}=B^{*}$ (see equation (3.8)), the Jacobian becomes


(3.5) $$\begin{array}{rl} J_{0} & =\left[\begin{array}{cc} \beta N^{*}-\omega^{*}-\gamma & (1-p)\beta N^{*}+\alpha^{*}\\ (1-c)\beta B^{*}+\omega^{*} & (1-c)(1-p)\beta B^{*}-\alpha^{*}-\gamma \end{array}\right]\;, \end{array}$$

where $\omega^{*}=\omega_{1}B^{*}+\omega_{3}$ and $\alpha^{*}=\alpha_{1}N^{*}+\alpha_{2}$ . Decomposing $J_{0}$ into the *epidemiological transmission* matrix [37]


$$T=\left[\begin{array}{cc} \beta N^{*} & (1-p)\beta N^{*}\\ (1-c)\beta B^{*} & (1-c)(1-p)\beta B^{*} \end{array}\right]$$

and the *transition* matrix


$$\Sigma =\left[\begin{array}{cc} -\omega^{*}-\gamma & \alpha^{*}\\ \omega^{*} & -\alpha^{*}-\gamma \end{array}\right],$$

the next-generation matrix is defined by $K=-T\Sigma^{-1}\;.$ Inverting −Σ gives


$$-\Sigma^{-1}=\frac{1}{(\gamma +\alpha^{*})(\gamma +\omega^{*})-\alpha^{*}\omega^{*}}\left[\begin{array}{cc} \alpha^{*}+\gamma & \alpha^{*}\\ \omega^{*} & \omega^{*}+\gamma \end{array}\right]\;,$$

and so


$$K=\left[\begin{array}{cc} \frac{\beta \,N^{*}\,\left(\alpha^{*}+\gamma +\left(1-p\right)\,\omega^{*}\right)}{\left(\gamma +\alpha^{*}\right)\,\left(\gamma +\omega^{*}\right)-\alpha^{*}\,\omega^{*}} & \frac{\beta \,N^{*}\,\left(\alpha^{*}+\left(1-p\right)\,\left(\omega^{*}+\gamma \right)\right)}{\left(\gamma +\alpha^{*}\right)\,\left(\gamma +\omega^{*}\right)-\alpha^{*}\,\omega^{*}}\\ \frac{\beta \,\left(1-c\right)\,B^{*}\,\left(\alpha^{*}+\gamma +\left(1-p\right)\,\omega^{*}\right)}{\left(\gamma +\alpha^{*}\right)\,\left(\gamma +\omega^{*}\right)-\alpha^{*}\,\omega^{*}} & \frac{\beta \,\left(1-c\right)\,B^{*}\,\left(\alpha^{*}+\left(1-p\right)\,\left(\omega^{*}+\gamma \right)\right)}{\left(\gamma +\alpha^{*}\right)\,\left(\gamma +\omega^{*}\right)-\alpha^{*}\,\omega^{*}} \end{array}\right].$$

Observing that det(K)=0 , we find the basic reproduction number of the BaD SIRS model is


(3.6) $$\begin{array}{rl} R_{0} & =tr(K)\\ & =\frac{\beta N^{*}(\alpha^{*}+\gamma +(1-p)\omega^{*})}{(\gamma +\alpha^{*})(\gamma +\omega^{*})-\alpha^{*}\omega^{*}}+\frac{\beta (1-c)B^{*}(\alpha^{*}+(1-p)(\omega^{*}+\gamma ))}{(\gamma +\alpha^{*})(\gamma +\omega^{*})-\alpha^{*}\omega^{*}}\\ & =\frac{\beta }{\gamma (\gamma +\alpha^{*}+\omega^{*})}\left(N^{*}(\alpha^{*}+\gamma +(1-p)\omega^{*})+(1-c)B^{*}(\alpha^{*}+(1-p)(\omega^{*}+\gamma ))\right)\;. \end{array}$$

To gain more insight into equation (3.6), we may rewrite it as


(3.7) $$\begin{array}{rl} R_{0} & =\frac{R_{0}^{D}}{\gamma +\alpha^{*}+\omega^{*}}\left(N^{*}(\alpha^{*}+\gamma +(1-p)\omega^{*})+(1-c)B^{*}(\alpha^{*}+(1-p)(\omega^{*}+\gamma ))\right)\\ & =R_{0}^{D}N^{*}+(1-c)R_{0}^{D}B^{*}-pR_{0}^{D}N^{*}\left(\frac{\omega^{*}}{\gamma +\alpha^{*}+\omega^{*}}\right)-p(1-c)R_{0}^{D}B^{*}\left(\frac{\omega^{*}+\gamma }{\gamma +\alpha^{*}+\omega^{*}}\right)\;, \end{array}$$

where


$$\frac{\omega^{*}}{\gamma +\alpha^{*}+\omega^{*}}$$

is the proportion of time we expect someone from N to spend in B and


$$\frac{\omega^{*}+\gamma }{\gamma +\alpha^{*}+\omega^{*}}=1-\frac{\alpha^{*}}{\gamma +\alpha^{*}+\omega^{*}}$$

is the proportion of time we expect someone from B to spend in B . Thus, we see that $R_{0}$ is made up of (i) the effect of individuals in N infecting individuals in N ( $R_{0}^{D}$ ) minus the effect of individuals in B infecting individuals in $N\left(pR_{0}^{D}\left(\frac{\omega^{*}}{\gamma +\alpha^{*}+\omega^{*}}\right)\right)$ , and (ii) the effect of individuals in N infecting individuals in B ( $(1-c)R_{0}^{D}$ ) minus the effect of individuals in B infecting individuals in $B\left(p(1-c)R_{0}^{D}\left(\frac{\omega^{*}+\gamma }{\gamma +\alpha^{*}+\omega^{*}}\right)\right)$ .

Finally, we note a few properties of interest. First, observe that when p=c=0 and behaviour has no effect on the epidemiology, $R_{0}$ reduces to $R_{0}^{D}$ . This reduction is readily seen in equation (3.7) since $N^{*}+B^{*}=1$ . Second, when p=c=1 and the protective behaviour is 100% effective at stopping transmission, the reproduction number reduces to


$$\frac{{ℛ}_{0}^{D}\,\left(N^{*}\,\left(\alpha^{*}+\gamma \right)\right)}{\left(\gamma +\alpha^{*}+\omega^{*}\right)}\leq {ℛ}_{0}^{D}.$$

From these, we note that $R_{0}$ is bounded above by $R_{0}^{D}$ . This suggests that the reproduction number from the standard SIRS model will be an overestimate to that suggested by the BaD SIRS model. Finally, by substituting $\omega^{*}N^{*}=\alpha^{*}B^{*}$ into equation (3.6), we see that $R_{0}$ is symmetric in the efficacy parameters p and c , that is, the basic reproduction number is blind to whether the behaviour is more protective for susceptible or for infectious individuals.

### 3.2. Steady states

**Figure 3.**
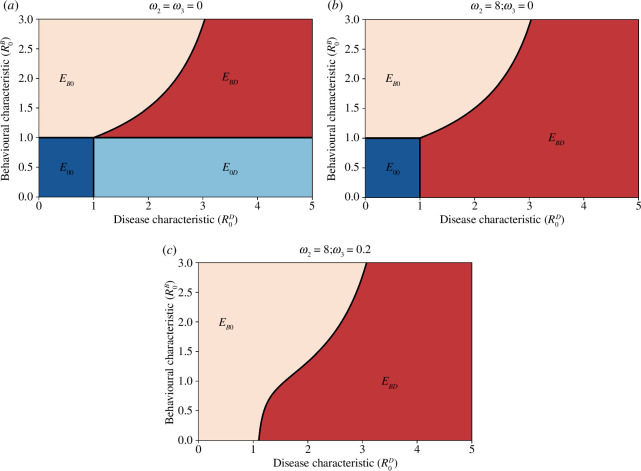
Possible equilibrium states from the BaD SIRS transmission model, where dark blue is the BaD-free equilibrium ( $E_{00}$ ), light blue is the behaviour-free-disease endemic equilibrium ( $E_{0D}$ ), light red is the behaviour endemic-disease free equilibrium ( $E_{B0}$ ) and dark red is the BaD endemic equilibrium ( $E_{BD}$ ). (*a*) No fear of disease or spontaneous uptake of behaviour ( $\omega_{2}=\omega_{3}$ = 0). (*b*) Fear of disease but no spontaneous uptake of behaviour ( $\omega_{2}=8,\omega_{3}=0$ ). (*c*) Both fear of disease and spontaneous uptake of behaviour ( $\omega_{2}=8,\omega_{3}=0.2$ ). Along the x -axis, we vary β through the disease characteristic $R_{0}^{D}$ , and along the y -axis, we vary $\omega_{1}$ through the behavioural characteristic $R_{0}^{B}$ . We have set p=c=0.5 , and all other parameters take values as in table 1. The vertical black line represents $R_{0}^{D}=1$ whereas the horizontal/curved black line shows $R_{0}=1$ . For a given behavioural characteristic $R_{0}^{B}$ , points on the horizontal/curved black line represent the critical values $R_{0}^{D*}$ of the disease characteristic where a disease endemic state becomes feasible.

There are four types of steady states for the BaD SIRS transmission model, which we characterize here. Regions in the $\left(R_{0}^{D},R_{0}^{B}\right)$ plane where the steady states exist and are stable are illustrated in figure 3.

#### 3.2.1. BaD-free steady state

In this steady state, we have $B^{*}=I^{*}=0$ and the steady-state vector is


$$E_{00}=\left(S^{*},0,0,0,0,0\right)=\left(1,0,0,0,0,0\right).$$

This state is only feasible when $\omega_{3}=0$ , $R_{0}^{D}<1$ and $R_{0}^{B}<1$ (figure 3*a*,*b*).

#### 3.2.2. Behaviour-free, disease-endemic steady state

In this steady state, $B^{*}=0$ and equations (2.4)–(2.9) reduce to the classic SIRS model. Hence, the steady state is given by


$$E_{0D}=\left(S^{*},I^{*},R^{*},0,0,0\right)=\left(\frac{\gamma }{\beta },\frac{\nu (\beta -\gamma )}{\beta (\gamma +\nu )},\frac{\gamma (\beta -\gamma )}{\beta (\gamma +\nu )},0,0,0\right).$$

This state is only feasible when $\omega_{2}=\omega_{3}=0$ , $R_{0}^{D}>1$ and $R_{0}^{B}<1$ (figure 3*a*).

#### 3.2.3. Behaviour endemic, disease-free steady state

This steady state occurs when $I^{*}=I_{N}^{*}+I_{B}^{*}=0$ and $B^{*}>0$ . This is given by


$$E_{B0}=\left(S_{N}^{*},0,0,S_{B}^{*},0,0\right)=\left(N^{*},0,0,B^{*},0,0\right),$$

where $N^{*}=1-B^{*}$ and $B^{*}$ solves


$$\begin{array}{rl} \dot{B} & =\omega (t)N-\alpha (t)B\\ & =(\alpha_{1}-\omega_{1})B^{2}-(\alpha_{1}-\omega_{1}+\omega_{3}+\alpha_{2})B+\omega_{3}\\ & =0\;. \end{array}$$

Writing


$$F\,\left(B^{*}\right)=\left(\alpha_{1}-\omega_{1}\right)\,{\left(B^{*}\right)}^{2}-\left(\alpha_{1}-\omega_{1}+\omega_{3}+\alpha_{2}\right)\,B^{*}+\omega_{3},$$

we see that $F(0)=\omega_{3}\geq 0$ and $F(1)=-\alpha_{2}\leq 0$ . Hence, there is a unique $B^{*}\in (0,1)$ with $F(B^{*})=0$ . If $\alpha_{1}=\omega_{1}$ then $F(B^{*})=0$ when


$$B^{*}=\frac{\omega_{3}}{\omega_{3}+\alpha_{2}}.$$

If $\alpha_{1}\neq \omega_{1}$ , then


(3.8) $$B^{*}=\frac{\alpha_{1}-\omega_{1}+\omega_{3}+\alpha_{2}-\sqrt{{\left(\alpha_{1}-\omega_{1}+\omega_{3}+\alpha_{2}\right)}^{2}-4(\alpha_{1}-\omega_{1})\omega_{3}}}{2(\alpha_{1}-\omega_{1})}\;.$$

Note that if $\omega_{3}=0$ , equation (3.8) can be rewritten as


$$B^{*}=\frac{\alpha_{1}+\alpha_{2}}{2\,\left(\alpha_{1}-\omega_{1}\right)}\,\left(\left(1-{ℛ}_{0}^{B}\right)-\left|1-{ℛ}_{0}^{B}\right|\right).$$

This demonstrates the dependence of $B^{*}$ on the behavioural characteristic $R_{0}^{B}$ , with $B^{*}=0$ when $R_{0}^{B}<1$ and $B^{*}>0$ when $R_{0}^{B}>1$ . The behaviour endemic, disease-free steady state is feasible when:

1. $\omega_{3}=0$ : $R_{0}^{B}>1$ and $R_{0}^{D}<R_{0}^{D*}$ for a critical value $R_{0}^{D*}$ calculated from $R_{0}=1$ (figure 3*a*,*b*), or;

2. $\omega_{3}\neq 0$ : $R_{0}^{D}<R_{0}^{D*}$ for a critical value $R_{0}^{D*}$ calculated from $R_{0}=1$ (figure 3*c*).

#### 3.2.4. BaD endemic state

The BaD endemic state is of the following form


$$E_{B\,D}=\left(S_{N}^{*},I_{N}^{*},R_{N}^{*},S_{B}^{*},I_{B}^{*},R_{B}^{*}\right),$$

and is more complex to determine. We derive $E_{BD}$ for when at least one of p≠0 or c≠0 is true; the case when p=c=0 , that is, when the protective behaviour does not affect the epidemiology at all is found in electronic supplementary material.

First note that, similar to deriving equation (3.8), given the endemic disease prevalence $I^{*}$ we get endemic behaviour prevalence $B^{*}$ as


(3.9) $$B^{*}=\frac{\alpha_{1}-\omega_{1}+\omega_{2}I^{*}+\omega_{3}+\alpha_{2}-\sqrt{{\left(\alpha_{1}-\omega_{1}+\omega_{2}I^{*}+\omega_{3}+\alpha_{2}\right)}^{2}-4(\alpha_{1}-\omega_{1})(\omega_{2}I^{*}+\omega_{3})}}{2(\alpha_{1}-\omega_{1})}\;,$$

when $\alpha_{1}\neq \omega_{1}$ or


$$B^{*}=\frac{\omega_{3}}{\omega_{2}\,I^{*}+\omega_{3}+\alpha_{2}}$$

when $\alpha_{1}=\omega_{1}$ . Hence, fixing $I^{*}$ determines $B^{*}$ , $N^{*}$ , $\alpha^{*}=\alpha_{1}B^{*}+\alpha_{2}$ and $\omega^{*}=\omega_{1}B^{*}+\omega_{2}I^{*}+\omega_{3}$ . Now, adding equations (2.6) and (2.9) gives


$$\dot{R}={\dot{R}}_{N}+{\dot{R}}_{B}=\gamma \,I-\nu \,R.$$

Setting this equal to 0 gives $R^{*}=\gamma I^{*}/\nu$ , and combining with the condition $S^{*}+I^{*}+R^{*}=1$ gives


$$S^{*}=1-\left(\frac{\gamma +\nu }{\nu }\right)\,I^{*},$$

that is, fixing $I^{*}$ also determines $S^{*}=S_{N}^{*}+S_{B}^{*}$ and $R^{*}=R_{N}^{*}+R_{B}^{*}$ . Now, if c=1 setting equation (2.8) to zero gives


$$I_{B}^{*}=\frac{\omega^{*}\,I^{*}}{\alpha^{*}+\omega^{*}+\gamma },$$

where we have used $I^{*}=I_{N}^{*}+I_{B}^{*}$ . Otherwise, equating equations (2.5) and (2.8) to zero gives


(3.10) $$\begin{array}{rl} \lambda^{*}S_{N}^{*} & =(\omega^{*}+\gamma )I^{*}-(\alpha^{*}+\omega^{*}+\gamma )I_{B}^{*}\;\text{, and} \end{array}$$

(3.11) $$\begin{array}{rl} \lambda^{*}S_{B}^{*} & =\frac{(\alpha^{*}+\omega^{*}+\gamma )I_{B}^{*}-\omega^{*}I^{*}}{\left(1-c\right)}\;, \end{array}$$

where $\lambda^{*}=\beta (I^{*}-pI_{B}^{*})$ . Adding equations (3.10) and (3.11) and solving for $I_{B}^{*}$ gives


(3.12) $$I_{B}^{*}=\frac{(1-c)(\beta S^{*}-\gamma )I^{*}+c\omega^{*}I^{*}}{\left(1-c)p\beta S^{*}+c(\alpha^{*}+\omega^{*}+\gamma \right)}\;.$$

Setting equation (2.9) to zero then gives


(3.13) $$R_{B}^{*}=\frac{\omega^{*}R^{*}+\gamma I_{B}^{*}}{\alpha^{*}+\omega^{*}+\nu }\;.$$

Equations (3.12) and (3.13) then determine all steady states given the final disease prevalence $I^{*}$ . Specifically, we have


$$\begin{array}{rl} S_{N}^{*} & =S^{*}-S_{B}^{*},\\ I_{N}^{*} & =N^{*}-S_{N}^{*}-R_{N}^{*},\\ R_{N}^{*} & =R^{*}-R_{B}^{*},\\ S_{B}^{*} & =B^{*}-I_{B}^{*}-R_{B}^{*},\\ I_{B}^{*} & =\frac{(1-c)(\beta S^{*}-\gamma )I^{*}+c\omega^{*}I^{*}}{\left(1-c)p\beta S^{*}+c(\alpha^{*}+\omega^{*}+\gamma \right)},\;\text{and}\\ R_{B}^{*} & =\frac{\omega^{*}R^{*}+\gamma I_{B}^{*}}{\alpha^{*}+\omega^{*}+\nu }\;. \end{array}$$

Finally, setting equation (2.4) to zero gives the relation


$$\left(\beta \,\left(I^{*}-p\,I_{B}^{*}\right)+\omega^{*}\right)\,S_{N}^{*}=\alpha^{*}\,S_{B}^{*}+\nu \,R_{N}^{*},$$

which fixes $I^{*}$ . The BaD endemic state is feasible when

1.
$\omega_{2}=\omega_{3}=0$ : $R_{0}^{B}>1$ and $R_{0}^{D}>R_{0}^{D*}$ for a critical value $R_{0}^{D*}$ calculated from $R_{0}=1$ (figure 3*a*), or;
2.
$\omega_{2}\neq 0$ or $\omega_{3}\neq 0$ : $R_{0}^{D}>R_{0}^{D*}$ for a critical value $R_{0}^{D*}$ calculated from $R_{0}=1$ (figure 3*b,c*).

## 4. Numerical explorations

To explore the BaD SIRS model, unless otherwise stated, we explore scenarios with epidemiological parameters inspired by influenza- and COVID-19-like illnesses. Scaling time so that the average infectious period is $\gamma^{-1}=1$ , this means investigating disease spread for β=1.5 (influenza-like) [38] and β=8.2 (COVID-19-like) [39]; we also fix $\nu^{-1}=40$ to indicate an immune period of 40 times the infectious period; for an infectious period of 7 days, this would correspond to an immune period of approximately 10 months, similar to that reported for the Omicron variant of COVID-19 [40]. To obtain the behavioural parameters, we scale the relevant parameters used by Funk *et al*. [19] and Perra & Vespignani [21] such that the infectious period is one. Specifically, we adopt the efficacy of behaviour parameters ( p,c ), social influence of behaviour ( $\omega_{1}$ ), spontaneous uptake of behaviour ( $\omega_{3}$ ) and spontaneous abandonment of behaviour ( $\alpha_{2}$ ) from Funk *et al.* [19], and the fear of disease ( $\omega_{2}$ ) and social abandonment of behaviour ( $\alpha_{1}$ ) from Perra & Vespignani [21]. The parameter values used are reported in table 1.

### 4.1. Behavioural concepts inform steady states

Influencing different components of the Health Belief Model can encourage the emergence of different steady-state solutions in the BaD SIRS model (figure 3). First, by encouraging the external cues to action, self-efficacy and perceived benefits of the behaviour at a population level (such that $\omega_{3}>0$ ) we observe there will always be protective behaviour in the system (figure 3*c*). In the absence of this (when $\omega_{3}=0$ ), we find that educating people on the perceived illness threat will ensure that protective behaviour is present in the system whenever disease is present endemically (figure 3*b*). Finally, by encouraging the social influence (external cues) of the protective behaviour, we can encourage behaviour to appear in the steady state of the system in a way that is spread as a social contagion as represented by the y -axis in each part figure of figure 3. Note that by targeting (reducing) the negative factors in the Health Belief Model—those that lead to behaviour abandonment—we also increase the social contagion aspect of protective behaviour in the model (equation (3.3)). Importantly, observe the nonlinear boundary between the states $E_{B0}$ and $E_{BD}$ , which represents the effect of behaviour on disease emergence. This boundary indicates that when protective behaviour is present in the steady state, the threshold of a disease to become endemic is reduced compared to a system with no protective behaviour.

### 4.2. Efficacy of protective behaviour

**Figure 4.**
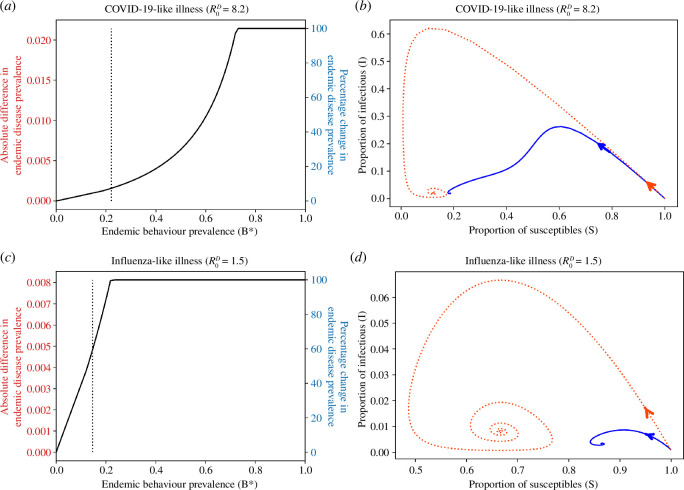
The difference in endemic disease prevalence between a population performing no health behaviours, compared to a population performing a fully protective health behaviour with p=c=1 for a COVID-19-like ( $R_{0}^{D}=8.4$ , (*a ,b*)) and an influenza-like ( $R_{0}^{D}=1.5$ , (*c ,d*)) illness. All other parameters take the values in table 1. (*a*) Difference in long-term disease prevalence between the two populations for a COVID-19-like illness ( $R_{0}^{D}=8.2$ ); the dotted vertical line is the proportion of the population performing the behaviour ( $B^{*}=0.22$ ) for the parameters given in table 1 and β=8.2 . (*b*) Phase plane of susceptible versus infectious individuals for a COVID-19-like illness ( $R_{0}^{D}=8.2$ ), where the orange dotted line shows the trajectory for a population with no health behaviour and the blue solid line shows the trajectory for a population with a fully protective health behaviour; the proportion of the population performing the behaviour in the long term is $B^{*}=0.22$ . (*c*) Difference in long-term disease prevalence between the two populations for an influenza-like illness ( $R_{0}^{D}=1.5$ ); the dotted vertical line is the proportion of the population performing the behaviour ( $B^{*}=0.15$ ) for the parameters given in table 1 and β=1.5 . (*d*) Phase plane of susceptible versus infectious individuals for an influenza-like illness ( $R_{0}^{D}=1.5$ ), where the orange dotted line shows the trajectory for a population with no health behaviour and the blue solid line shows the trajectory for a population with a fully protective health behaviour; the proportion of the population performing the behaviour in the long term is $B^{*}=0.15$ .

Protective behaviours such as facemask wearing and physical distancing are important to help reduce the spread of respiratory diseases, but their effect on endemic disease prevalence has been unclear. To investigate this, we compare the endemic disease prevalence for two populations: the first with no protective behaviour ( p=c=0 ), and the second with a fully protective behaviour ( p=c=1 ). This captures the most extreme case of the effect of a health-protective behaviour on endemic disease prevalence. To achieve a 20% reduction in endemic disease prevalence for a highly transmittable disease ( $R_{0}^{D}=8.2$ , such as COVID-19) we need at least 43% of the population performing the fully protective behaviour in the long term; this proportion increases to 73% performing the behaviour in the long term to eliminate the infection (figure 4*a*). Comparatively, for a less transmittable infection ( $R_{0}^{D}=1.5$ , such as influenza) we need at least 8% of the population performing the behaviour in the long term to see a 20% reduction in infection prevalence, and at least 22% performing the behaviour to eradicate the infection (figure 4*c*).

Observing figure 4*a,c* suggests that we need a reasonably large proportion of the population performing a fully protective behaviour to notice a decrease in the long-term disease prevalence in the community. However, the path to the endemic disease prevalence differs greatly between a population with a protective behaviour compared to one without a protective behaviour. Figure 4*b,d* show the phase planes for susceptible and infectious individuals for a COVID-19- and influenza-like infection, respectively. Notice that the steady-state behaviour might be similar in the two populations (particularly for a COVID-19-like infection), but the transient trajectory of the infection in the communities varies greatly. Specifically, for both the COVID-19-like and influenza-like infections, the impact for the disease on the protected population is substantially less than in the non-protected population, where impact is measured by the peak prevalence of infection over time.

### 4.3. Targeted interventions on controlling disease dynamics

**Figure 5.**
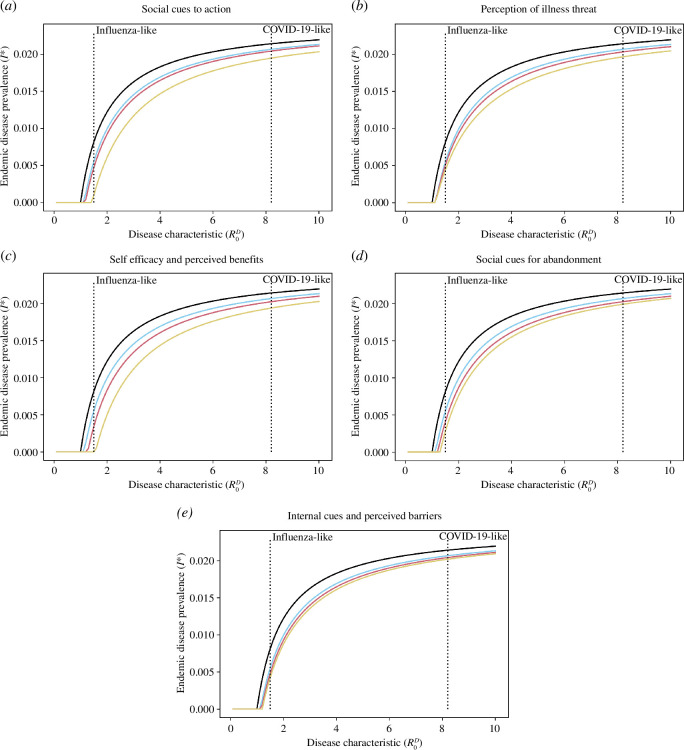
The effect of intervention strategies targeted at components of the Health Belief Model on endemic disease prevalence. The x -axis is the disease characteristic $R_{0}^{D}$ , and the y -axis is the endemic disease prevalence. The vertical dotted lines represent an influenza-like disease characteristic ( $R_{0}^{D}=1.5$ ) and a COVID-19-like disease characteristic ( $R_{0}^{D}=8.2$ ). The lines show a no-behaviour SIRS model (black), a base-line BaD SIRS model (light blue), a good intervention doubling (or halving) the effect (red), and an excellent intervention quadrupling (or quartering) the effect (yellow). (*a*) Social cues to action ( $\omega_{1}$ ). (*b*) Perception of illness threat ( $\omega_{2}$ ). (*c*) Self-efficacy and perceived benefits of behaviour ( $\omega_{3}$ ). (*d*) Social cues for abandonment ( $\alpha_{1}$ ). (*e*) Internal cues to action and perceived barriers to behaviour ( $\alpha_{2}$ ). Baseline parameter values are defined in table 1 with p=c=0.5 .

By incorporating the Health Belief Model into our BaD SIRS model, we gain the capacity to investigate non-pharmaceutical interventions targeted at components of the Health Belief Model and their effects on disease endemicity. Targeted interventions and educational campaigns around the components of the Health Belief Model make it possible to influence the likelihood that individuals will perform a given protective behaviour. Here, we explore a good intervention (doubling the effect) and an extremely successful intervention (quadrupling the effect). For drivers of behaviour abandonment (perceived barriers and cues to action), we consider the good intervention as halving the coefficient, and the extreme intervention of quartering the coefficient. The results are shown in figure 5.

First, note that the presence of behaviour in the system (light blue line) compared to the standard SIRS model (black line) suggests a smaller endemic disease prevalence. Furthermore, with the exception of perception of illness threat and internal cues to action/perceived barriers, we observe that a good intervention (red line) has a minimal effect on the endemic disease prevalence in comparison to the baseline model (light blue line). An extremely successful intervention (yellow line) has a more substantial effect for a less infectious disease ( $R_{0}^{D}<5$ ), but the differences between a good intervention (red line) and an extremely successful intervention (yellow line) are less noticeable for highly infectious diseases (with the exception of perception of illness threat and internal cues to action/perceived barriers to the behaviour). Overall, for mildly infectious diseases targeting social cues to action and self-efficacy/perceived benefits of the behaviour may have a profound impact on the prevalence of endemic disease as they have the potential to eliminate the infection (by bringing $R_{0}<1$ ). However, for more highly infectious diseases, it can also prove beneficial to target the perception of illness threat to help reduce disease prevalence.

## 5. Discussion

We presented a BaD SIRS model to exemplify a structured approach to incorporating behavioural science theories into standard epidemiological models. Using our model, we have identified semi-analytic solutions of the steady states of the system and used these to explore the impact of behaviour on endemic disease prevalence. We have highlighted how we can explore the effects of non-pharmaceutical interventions on the disease prevalence expected in a community, where the interventions target-specific aspects of behaviour change (e.g. the perception of illness threat).

The BaD SIRS model generalizes previously studied models for behaviour–disease interactions [19–22] by combining the behavioural parameters and providing a behavioural science motivation for why these parameters are reasonable. This approach adds validity to the behaviour change parameters that have previously been considered. Using our model, we found similar results to [19] and [20] that show that the presence of protective behaviour reduces the infection invasion threshold. We similarly demonstrated that increased spontaneous uptake of the behaviour (the so-called awareness campaigns of [19] and [20]) can reduce disease prevalence in the community. However, our results also suggest that targeting social cues to action and perception of illness threat, particularly for highly contagious diseases, can provide a notable reduction in endemic disease prevalence.

Importantly, our BaD transmission framework opens the door for new transdisciplinary research in BaD modelling. This has consequences for both modelling of infectious diseases and policymaking. Incorporating human behaviour into disease transmission models is a vital step towards obtaining more realistic ‘business-as-usual’ models for baseline modelling of new infectious diseases. As demonstrated here, BaD models allow us to explore the effects of interventions for reducing disease spread that are more targeted towards human behaviour. This will allow the co-design of more impactful non-pharmaceutical interventions for disease control.

However, there are some pivotal steps to be taken before BaD transmission models can be translated into real-world impact. One major roadblock to applying BaD transmission models to real-world problems is the availability of appropriate data sources to fit the models [9,10]. Even in the simple case of the BaD SIRS model, fitting the behavioural parameters is non-trivial. Another issue is adequate model specification, both in the infection components and the behavioural components. We have presented one of the most basic disease structures as an exemplary approach, but more complex infection dynamics can and should be considered. Likewise, we have considered only a binary stratification of behaviour in this article with behavioural transitions motivated by the Health Belief Model, which like all behavioural models has limitations, such as a lack of consideration of the emotional influences on decision-making [31,41]. Behaviours can instead be quantified at various levels of specificity with linear, continuous variables largely favoured by behavioural scientists. Similarly, no single theoretical health behaviour model is considered superior to any other, although most capture socio-cognitive predictors in a regressive model just like the Health Belief Model.

For future work, we suggest choosing the most appropriate behavioural model and structure, as well as appropriate assumptions on the components of the behavioural model, to fit the target behaviour, infection and questions of interest. To further overcome these challenges, new studies and models need to be co-designed by behavioural scientists and epidemiologists to gain better insight into the most appropriate model and how to collect the right data to fit the model. A related limitation of our model is that our population-level strata structure assumes homogeneity of behaviour, whereas behaviour and effects of behavioural interventions are known to be heterogeneous [42]. Since such heterogeneity can result in different dynamics (e.g. [43–45]), agent-based or network theory approaches should be used in the future to explore the effect of this heterogeneity on the interface between behaviour and infection. Other areas of future research include investigating more complex behavioural transitions such as time-delayed information retrieval and memories of behaviour to better capture behavioural dynamics in the system, and incorporating other aspects of human behaviour such as mobility to understand the impacts this has on behavioural decision-making.

Understanding the human behaviour aspect of how infections spread provides better insight into the stresses we can expect on our public health systems in the face of a new pandemic. Our work here has provided a structured approach for incorporating the rich theory from behavioural science into standard infection transmission models in a way that captures behavioural uptake in a more realistic manner and identifies areas for targeted interventions to increase positive health behaviours in response to an epidemic. By fusing behavioural sciences and epidemiological modelling, our BaD modelling framework can be used to investigate more realistic ‘business-as-usual’ models of how we can expect infections to spread through a community. By developing these better ‘business-as-usual’ models for infection transmission, we will be in a better position to inform policy around public health practices. This will not only have consequences for the economic and public health response to future epidemics, but will increase public trust in our modelling processes.

## Data Availability

The code has been published in Zenodo [46]. Electronic supplementary material is available online at [47].
